# Dietary exposure to acrylamide of university students in Ningxia of Northwest China and the effect on their neurobehavioral performance and oxidative stress in serum

**DOI:** 10.1002/fsn3.3101

**Published:** 2022-10-17

**Authors:** Qinghan Gao, Yanzhong Xue, Xiao Yuan, Hua Gao, Chunsen Wu

**Affiliations:** ^1^ School of Public Health and Management Ningxia Medical University Yinchuan China; ^2^ Department of Pharmacy General Hospital of Ningxia Medical University Yinchuan China; ^3^ School of Food Science & Engineering Yangzhou University Yangzhou China

**Keywords:** acrylamide, risk, neurobehavioral performances, oxidative stress

## Abstract

This study aims to investigate dietary exposure to acrylamide (AA) and also make an assessment of its effect on the neurobehavioral performance and oxidative stress in the serum of university students in Ningxia. The place primarily consists of arid, dry desert, and wheat‐based foods are the staple food there. A total of 803 university students in Ningxia participated in this cross‐sectional study. Diet intake of AA was estimated with FFQ. The AA risk was calculated as margin of exposure (MOE) values. NCTB questionnaires were used to assess neurobehavioral performance. The serum oxidative stress levels of the university students were measured as GSH, MDA, and SOD. The mean for AA exposure of university students was 0.515 μg kg^−1^ bw day^−1^. The highest contributor was traditional Chinese grain products, representing 34.71% of the total daily AA intake. Followed were deep‐fried potato products, traditional Western grain products, soft drinks, and nuts, which accounted for 23.87%, 16.59%, 11.15%, and 11%, respectively. The median AA exposure were 480 (BMDL_10_ = 0.18 mg kg^−1^ bw day^−1^) and 827 (BMDL_10_ = 0.31 mg kg^−1^ bw day^−1^), respectively. The results indicated that diet AA may have an effect on the emotional status and neurobehavior among this population. We observed no significant differences in oxidative stress under the three levels of AA exposure (*p* > .05). It suggests a health concern for university students in Northwest China that should get society's attention.

## Graphical abstract

This study aims to investigate dietary exposure to acrylamide (AA) of university students in the Northwest China and the effect on their neurobehavioral performance and oxidative stress in serum. The results indicated that diet AA may affect the emotional status and neurobehavioral performance in this population. We observed no significant differences in oxidative stress under the three levels of AA exposure (*p* > .05). It suggests that there is a health concern with respect to university students in Northwest China.

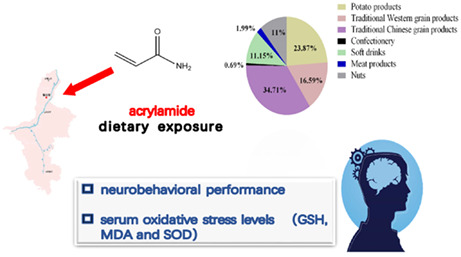

## 1 INTRODUCTION

A highly processed diet not only provides nutrients for humans but also formed ingredients during the period of process and storage. Compounds formed during food production may exert carcinogenic and neurotoxic effects (Koszucka & Nowak, 2019). Among these compounds, acrylamide (AA) was found recently in food by scientists from Sweden (Mottram et al., 2002). AA is generated during the stage of the Maillard reaction, resulting from the interaction between asparagine and reducing sugars in heat‐treated starchy foods (Hodge, 1953). French fries (689–693 μg kg^−1^), roasted coffee (225–231 μg kg^−1^), and soft bread (27–37 μg kg^−1^) are reported to be the three major contributors to AA exposure in adults (EFSA, 2011).

Previous studies have found that teenagers easily consume more snacks like cookies, potato crisps, or French fries compared with other age groups. Higher AA intake of this age group was due to their higher consumption of snacks with higher levels of AA. Meanwhile, it is easy to buy those products in university shops and fast food restaurants (Kowalska et al., 2017). Several studies have suggested that there is a relationship between AA exposure and the risk of cancer in humans (Adani et al., 2020). MOEs have been calculated for the induction of mammary tumors in rats (BMDL_10_ = 0.31 mg AA kg^−1^ bw), which are among 78–310 (World Health Organization, 2011). Other research groups have given the corresponding application of MOE values to substances in food that are genotoxic and carcinogenic (Carthew et al., 2010). Among infants and adults, the dose–response modeling of the data for hepatocellular tumors gives a BMDL10 of 1.23 mg kg^−1^ day^−1^, and MOEs of between 750 and 4300 for exposures to furan that is commonly found in foods.

Studies have shown that AA exposure has been related to the risk of neurotoxicity. However, the effects of consistent dietary AA exposure on neurobehavioral performance are still researched less. Changes in neurobehavior are considered to be the early indicators of impairment in the nervous system and marks of the potential neurotoxicity of the existing substances (Moser, 1990). The World Health Organization Neurobehavioral Core Test Battery (NCTB) is the most commonly used human neurobehavioral test as the screening tool for detecting neurotoxic chemicals exposures and identifying their harmful effects (Anger, 2014). Previous studies have shown AA exposure could increase oxidative stress in animals. However, population studies make the assessment of the association between AA and oxidative stress scarce.

In Western countries, the daily intake of AA by adults is 1–4 μg kg^−1^ bw (EFSA, 2011). To the best of our knowledge, the data about the dietary intake of AA among university students in China, especially in the northwest of China, are lacking. Furthermore, the main sources of dietary AA are different among Asian areas and Western countries. In Japan, coffee, green tea, and confectioneries are the main sources of AA. But for the Western countries, potato and wheat‐based foods, as well as coffee, are the three richest sources of AA (Kotemori et al., 2018). In addition, Ningxia is located in the northwest of China, and wheat‐based foods are the staple food there. Therefore, it is necessary to examine the intake and the influence of AA in various regions with different dietary habits.

The present study was to assess the dietary exposure to AA of university students in Ningxia, identify food items that are the main sources of AA exposure, as well as to determine the relationship between AA exposure and neurobehavioral performance and oxidative stress levels. MOE values were applied to assess the risk related to dietary exposure to AA.

## 2 MATERIALS AND METHODS

### 2.1 Participants

The study was conducted from October to December 2019 in 803 nonsmoking young people, and 67.6% were girls. Their ages were between 17 and 26 years. Subjects were excluded from this group population for the following reasons: experiencing depression during the past 3 months, encephalopathy, family with neuropsychological disorders or personal, autoimmune diseases, or ingested medication 2 weeks before the survey which could have an effect on the nervous system. All the participants lived in Ningxia. The research staff explained the purpose of the study and the procedures to the students who expressed their interest in participating in this study. Ethical clearance was obtained from Ningxia Medical University. Students' lack of consent, long‐term absences from university with illness, or participation in some contests or competitions were rejected access to the research group.

### 2.2 Dietary questionnaire

Firstly, with a valid and reproducible food frequency questionnaire, the daily intake of AA food sources was estimated. The questionnaire documented the frequency of consumption of foods and drinks over the preceding month. Standardized portions of real food models were provided to help participants to better describe the amounts of foods and drinks they consumed. These models correspond to a known volume/weight and are based on everyday tableware.

### 2.3 Food consumption data

The data on food consumption were obtained face‐to‐face by trained interviewers. Foods items included are mainly products with a certain amount of AA and consumed frequently by the subjects. AA exposure was calculated according to Food Consumption Data of the Fifth Chinese Total Diet Study 2009 (Gao et al., 2016), which is the most recent record of dietary consumption at a national level.

### 2.4 Risk assessment of AA exposure

MOE represents the distance between the dose of benchmark in rats associated with 10% tumor incidence and the estimated average exposure in humans. The values of MOE were calculated by comparing AA exposure values against BMDL_10_ values (0.18 mg kg^−1^ bw day^−1^ and 0.31 mg kg^−1^ bw day^−1^, respectively).

### 2.5 Neurobehavioral function tests

Neurobehavioral function was performed with NCTB consisting of different questionnaires on mood conditions and a series of behavior measurements. The states of mood include the following six items: anger–hostility (AAMSA), fatigue–inertia (AAMSF), depression–dejection (AAMSD), vigor–activity (AAMSV), confusion–bewilderment (AAMSC), and tension–anxiety (AAMST).

Two independent performing tests are as follows: (1) digit symbol for reflecting the speed of information processing within 90 s; (2) pursuit aiming for reflecting the speed and accuracy of manual operation.

All the students participated in the process of the survey in the morning in a quiet room. All interviewers were trained before the investigation with the NCTB Guidance. Each test was performed individually and scored by an interviewer.

### 2.6 Measurement of the levels of SOD, GSH and MDA

To determine the biomarkers of oxidative stress, serum was collected from whole blood after centrifugation at 4000 rpm at 4°C for 10 min. SOD, GSH and MDA of the serum were determined according to the manufacturer's instructions of commercial kits. The results of MDA, GSH and SOD were expressed as nmol ml^−1^, μmol g^−1^ and U ml^−1^, respectively.

### 2.7 Statistical analysis

Data in this study were double entered with EpiData 3.1. Descriptive statistics were applied to calculate for the AA intake of the consumption foods and the dietary AA exposure of those participants. Non‐normal distribution data were analyzed after data transformation. Spearman correlation analysis was used for analyzing the relationships between dietary exposure to AA and neurobehavioral function, as well as oxidative stress. *p* ≤ .05 was considered to be at a level of statistical significance. Data analysis was done with SPSS 22 (IBM Corp., Armonk, NY, USA).

## 3 RESULTS

### 3.1 Population description

Compared to older age groups, university students can easily form good eating habits and are easily exposed to AA at the same time. In total, there were 543 girls and 260 boys participated in this study. The rate of recruitment was about 11.8%, i.e., 803 participants of 6804 undergraduate students were contacted. Most of them were of Ningxia origin.

### 3.2 Food groups in food rations

**FIGURE 1:**
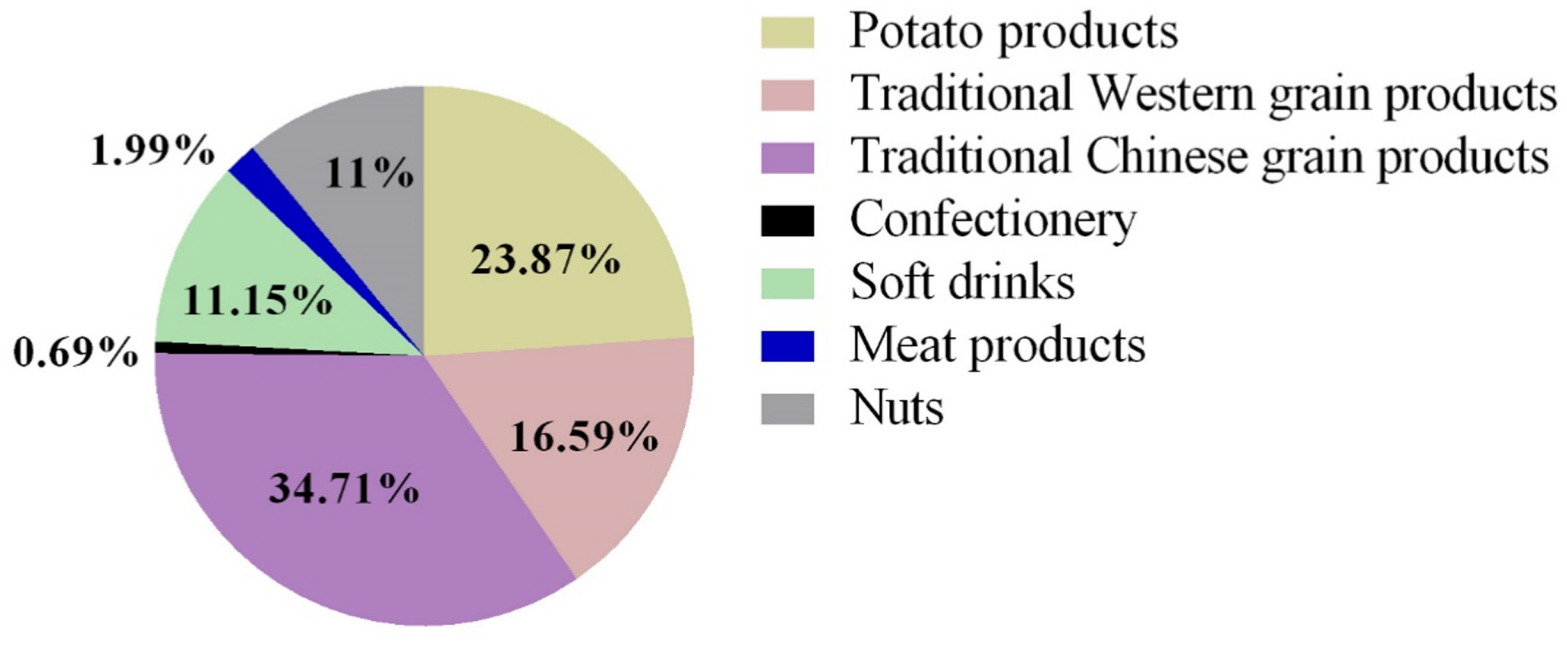
Contribution (%) of the main food groups to the dietary exposure to acrylamide (AA) of university students in Northwest China.

**FIGURE 2:**
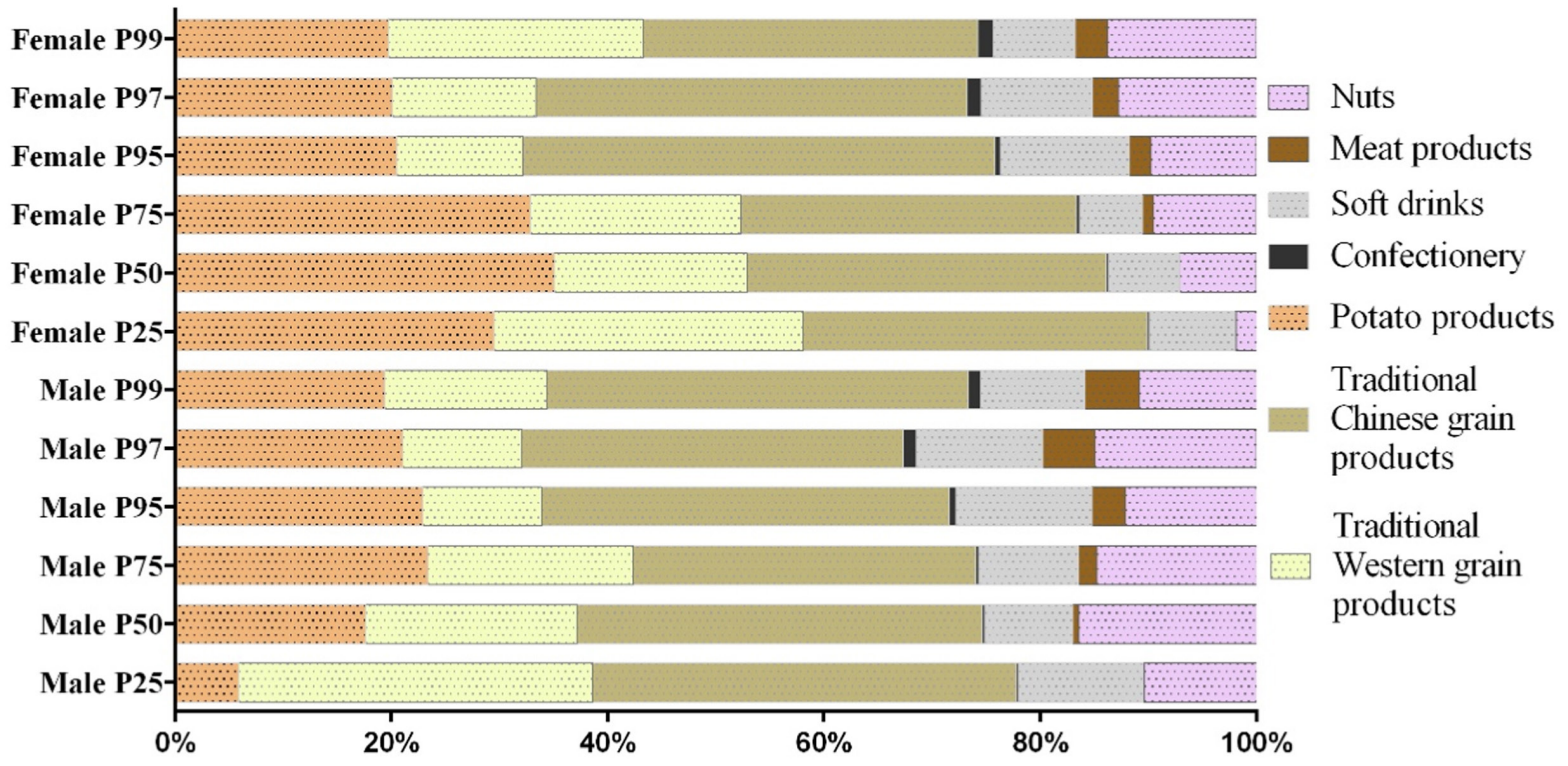
Acrylamide (AA) intake values in different food groups with sex classification.

Daily intake of AA from consumption with different food groups of girls and boys is presented in Figure 1. The results showed that food groups of AA exposure were similar in girls and boys. Daily AA intake was mainly from the consumption of traditional Chinese grain products, potato products, and traditional Western grain products. And the higher level of AA exposure, the more intake of traditional Chinese grain products and potato foods in boys, and the higher intake of traditional Western grain products in girls (Figure 2). Traditional Chinese grain products represented 34.71% of total daily AA intake as the highest contribution. Deep‐fried potato products, traditional Western grain products, soft drinks, and nuts accounted for 23.87%, 16.59%, 11.15%, and 11% of daily intake, respectively. Altogether, these four items represented 97.32% of AA intake daily.

### 3.3 Dietary exposure to AA of girls and boys

The characteristics of AA intake in girls and boys were summarized in Tables 1 and 2. The overall mean for AA exposure of university students was 0.515 μg kg^−1^ bw day^−1^. The median intake for boys is estimated to be 0.483 μg kg^−1^ bw day^−1^. The 25th percentile of intake is 0.207 μg kg^−1^ bw day^−1^ while the 99th percentile is 1.711 μg kg^−1^ bw day^−1^. As a whole, the girls’ dietary intake AA was higher compared with that of boys. More specifically, the median intake for girls is 0.53 μg kg^−1^ bw day^−1^.

**TABLE 1:** Dietary intake of acrylamide of different genders in university students.

	Dietary intake of acrylamide μg kg^−1^ bw day^−1^		
	Total	Male	Female
*n*	803	260	543
Mean	0.515	0.483	0.530
P25	0.207	0.184	0.220
P50	0.375	0.358	0.380
P75	0.672	0.657	0.687
P90	1.214	1.105	1.262
P95	1.493	1.414	1.517
P97	1.633	1.500	1.639
P99	1.711	1.660	1.740

**TABLE 2:** Dietary intake (DI) of acrylamide in university students.

	Low‐exposure group DI < 0.4 μg kg^−1^ bw day^−1^	Middle‐exposure group 0.4 < DI < 0.8 μg kg^−1^ bw day^−1^	High‐exposure group DI > 0.8 μg kg^−1^ bw day^−1^
Mean	0.224	0.560	1.252
P25	0.143	0.456	1.017
P50	0.222	0.554	1.217
P75	0.311	0.659	1.493
P90	0.366	0.726	1.663
P95	0.378	0.766	1.711
P97	0.389	0.781	1.749
P99	0.397	0.797	1.780

### 3.4 Risk assessment

MOE values are shown in Table 3. For median AA exposure, the MOE values were 480 (BMDL_10_ = 0.18 mg kg^−1^ bw day^−1^) and 827 (BMDL_10_ = 0.31 mg kg^−1^ bw day^−1^). The MOE value was all under 10,000 and even in some cases were below 200.

**TABLE 3:** Risk assessment of dietary exposure to acrylamide in university students.

	Mean	P25	P50	P75	P95	P97	P99
Dietary Exposure to Acrylamide μg kg^−1^ bw day^−1^	0.515	0.207	0.375	0.672	1.493	1.633	1.711
MOE (BMDL_10_) = 0.18 mg kg^−1^ bw day^−1^	350	870	480	268	121	110	105
MOE (BMDL_10_) = 0.31 mg kg^−1^ bw day^−1^	602	1498	827	461	208	190	181

### 3.5 Neurobehavioral function

Neurobehavioral test scores are listed in Tables 4 and 5. For mood states, there were significantly higher AAMST and AAMSD scores for students from the high AA exposure group compared with the low‐exposure and middle‐exposure students (*p* < .05). AAMSV scores were significantly lower in low‐exposure group compared to the high‐AA exposure group (*p* < .05). There were no significant differences in the other mood state scores of the three groups (*p* > .05). In the digit symbol test, digit symbol scores were negative with the levels of AA exposure. But there were no significant differences among the three groups in other neurobehavioral performance scores (*p* > .05).

**TABLE 4:** The profile of mood states of different acrylamide exposure groups in university students.

Mood States	Low‐exposure group DI < 0.4 μg kg^−1^ bw day^−1^	Middle‐exposure group 0.4 < DI < 0.8 μg kg^−1^ bw day^−1^	High‐exposure group DI > 0.8 μg kg^−1^ bw day^−1^
Tension–Anxiety (AAMST)	18.55 ± 4.86	19.31 ± 5.12^a^	19.77 ± 4.59^a^
Depression–Dejection (AAMSD)	27.52 ± 9.13	29.05 ± 9.94^a^	29.98 ± 8.60^a^
Anger–Hostility (AAMSA)	21.44 ± 6.29	21.19 ± 6.63	21.91 ± 6.17
Fatigue–Inertia (AAMSF)	14.82 ± 4.48	15.96 ± 4.62	15.47 ± 4.27
Confusion–Bewilderment (AAMSC)	15.69 ± 3.41	16.86 ± 3.84	16.49 ± 3.88
Vigor–Activity (AAMSV)	23.85 ± 5.54	23.34 ± 5.54	23.30 ± 5.09^a^

^a^ Compared with low‐exposure group at *p <* .05.

**TABLE 5:** The neurobehavioral test of different acrylamide exposure groups in university students.

Test items	Low‐exposure group DI < 0.4 μg kg^−1^ bw day^−1^	Middle‐exposure group 0.4 < DI < 0.8 μg kg^−1^ bw day^−1^	High‐exposure group DI > 0.8 μg kg^−1^ bw day^−1^
Digit Symbol			
Total Digit Symbol	66.30 ± 11.57	65.80 ± 10.05	65.53 ± 11.74
Pursuit Aiming			
Correct Pursuit Aiming	143.77 ± 29.18	143.06 ± 35.89	143.08 ± 26.32
Error Pursuit Aiming	6.53 ± 7.55	7.16 ± 10.10	8.60 ± 14.20
Total Pursuit Aiming	152.32 ± 31.71	154.09 ± 57.71	149.64 ± 27.73

### 3.6 Oxidative stress

Neurotoxic effects of dietary AA have increasing evidence, but its specific mechanism is still unclear. We observed no significant differences in oxidative stress biomarkers under the three levels of AA exposure (*p* > .05). But the present results suggested AA exposure may increase oxidative stress at high intakes (Table 6).

**TABLE 6:** The oxidative stress of different acrylamide exposure groups in university students.

	Low‐exposure group DI < 0.4 μg kg^−1^ bw day^−1^	Middle‐exposure group 0.4 < DI < 0.8 μg kg^−1^ bw day^−1^	High‐exposure group DI > 0.8 μg kg^−1^ bw day^−1^
GSH (μmol g^−1^)	75.62 ± 8.76	75.42 ± 8.17	82.15 ± 7.55
MDA (nmol ml^−1^)	4.36 ± 1.18	4.31 ± 1.24	4.54 ± 1.36
SOD (U ml^−1^)	189.60 ± 19.47	188.34 ± 18.41	186.33 ± 18.93

## 4 DISCUSSION

The International Agency for Research on Cancer (IARC) in 1994 classified AA as “possibly carcinogenic to humans” (Group 2A) based on the probability of industrial exposure to AA and its daily intake from water and tobacco smoke (IARC, 1994). Traditional Chinese grain products consumed in a relatively large amount in daily life contribute significantly to the AA intake. Snack foods such as biscuits and bread also substantially attributed to this food group. Mestdagh et al. (2007) suggested that biscuits, French fries, bread, as well as chocolate were the four main contributors to AA intake at Ghent University (Belgium) (Mestdagh et al., 2007). Their research suggested a balanced diet with full nutrients may contribute to a decreased AA intake through an intervention group. In another previous study, their results showed that AA was detected from 144 tested food samples collected from the whole of China accounting for 43.7% of selected foods (Zhou et al., 2013). Among the Chinese population, the mean of the estimated dietary exposure to AA was 0.286 μg kg^−1^ bw day^−1^. As we know, different from Western diets, the Chinese diet is mainly derived from plants including cereals and vegetables (Matthys et al., 2005). Therefore, cereals, potatoes, legumes nuts, vegetable products, and meats were the main contributing food groups to AA exposure in the general population in China. In the USA, dietary AA mainly comes from potato chips, French fries, bread, cereals, biscuits/cookies, home fries, fried pastries, and other salty snacks (Friedman, 2003). In European countries, most of the dietary intake AA is from bread, crisp bread, rusks, coffee, and potatoes consumption (Freisling et al., 2013). In the Hubei Province of China, the highest concentration of AA was found in the potato sample (211.8 mg kg^−1^), followed by Inner Mongolia, where the sugar sample with the higher level of AA (180.6 mg kg^−1^), and the potato sample was 121.5 mg kg^−1^ in Sichuan Province (Gao et al., 2016).

Dietary exposure to AA is determined not only by the level of AA in food but also by the consumption amount of food. Some foods could be a significant source of AA, although they are lower in AA. Because they are staple foods in our diet and are consumed in large quantities in our daily life for the population in China (Xu et al., 2014).

Dietary intake (DI) of acrylamide in university students was classified into the following three grades: low‐exposure group (DI < 0.4 μg kg^−1^ bw day^−1^), middle‐exposure group (0.4 < DI < 0.8 μg kg^−1^ bw day^−1^), and high‐exposure group (DI > 0.8 μg kg^−1^ bw day^−1^) (FAO，WHO, 2002). For the low‐exposure group, the mean was 0.224 μg kg^−1^ bw day^−1^. For the middle‐exposure group and the high‐exposure group, the values were 0.56 μg kg^−1^ bw day^−1^ and 1.252 μg kg^−1^ bw day^−1^, respectively. Recently, the average AA intake in the general population is in the range 0.3–2.0 μg kg^−1^ bw day^−1^ as estimated by JECFA. JECFA also reported the mean dietary intake of AA to be between 0.2 and 1.0 μg kg^−1^ bw day^−1^ in adults, and the highest intake lies in the range 0.6–1.8 μg kg^−1^ bw day^−1^. For high percentile (90th–97.5th) consumers, the estimates are among 0.6–3.5 μg kg^−1^ bw day^−1^ (FAO/WHO, 2005). It is the same with previous research that boys also consumed more French fries than girls (Matthys et al., 2005). From the data, it was clear that much higher AA exposure is found in part of some university students.

MOE is applied for the risk assessment for genotoxic compounds with no tolerable daily intake recommended. When the MOE is above 10,000 for a compound with genotoxic and carcinogenic, it is considered to be a low health concern (Pedreschi et al., 2014). In the present study, MOE values are of great food health concern for some university students, although they are higher than JECFA reported (310 and 180). For the Chinese general population, MOEs of AA exposure were 973 (BMDL_10_: 0.31 mg kg^−1^ bw day^−1^) and 565 (BMDL_10_: 0.18 mg kg^−1^ bw day^−1^) reported in the fifth Chinese Total Diet Study. Therefore, lower MOE values for university students in Ningxia located in the northwest of China indicate that great concern should be given to these students who consume carbohydrate‐rich heated foods with higher levels of AA. In addition, knowledge of the occurrence and formation of AA should be known among these populations (Kowalska et al., 2017).

The present results suggested that the emotional status and neurobehavioral test performance in this population group may be affected by AA diet exposure. Other previous studies indicated that oxidative stress induced by AA is mainly through increased concentrations of ROS and decreased antioxidant defense enzyme levels in animals (Nowak et al., 2020; Pan et al., 2018).

Overall, this study evaluated the relationship between AA exposure and neurobehavioral function as well as the oxidative stress biomarkers in a representative population‐based sample for the first time. This study reflects the possibility that high levels of AA may increase oxidative stress in the process of some biological activities. The whole society should be aware of the potential neurotoxic effects of dietary AA exposure among university students. In line with JECFA and also based on current results, we should make efforts to reduce exposure to this genotoxic chemical (World Health et al., 2011). Firstly, teenagers can be discouraged to consume foods containing high AA; and secondly, AA formation during the food process in the university cafeteria should be reduced through mitigation strategies.

## 5 CONCLUSIONS

Diet AA may have an effect on the emotional status and neurobehavioral performance in this population. We observed no significant differences in oxidative stress under the three levels of AA exposure (*p* > .05). This study suggested that a health concern for university students should be drawn in Northwest China.

## CONFLICT OF INTEREST

The authors have declared no conflicts of interest for this article.

## ETHICS STATEMENT

Ethical clearance was obtained from Ningxia Medical University.
